# Magnetism in icosahedral quasicrystals: current status and open questions

**DOI:** 10.1088/1468-6996/15/4/044801

**Published:** 2014-07-02

**Authors:** Alan I Goldman

**Affiliations:** Ames Laboratory US DOE and the Department of Physics and Astronomy, Iowa State University, Ames, IA 50011, USA

**Keywords:** quasicrystals, rare earth magnetism, frustration, spin glass transition, quantum critical behavior, magnetic ordering, neutron scattering

## Abstract

Progress in our understanding of the magnetic properties of *R*-containing icosahedral quasicrystals (*R* = rare earth element) from over 20 years of experimental effort is reviewed. This includes the much studied *R*-Mg-Zn and *R*-Mg-Cd ternary systems, as well as several magnetic quasicrystals that have been discovered and investigated more recently including Sc-Fe-Zn, *R*-Ag-In, Yb-Au-Al, the recently synthesized *R*-Cd binary quasicrystals, and their periodic approximants. In many ways, the magnetic properties among these quasicrystals are very similar. However, differences are observed that suggest new experiments and promising directions for future research.

## 1. Introduction

Quasicrystals, discovered by Dan Shechtman in 1982, are distinguished by the presence of sharp Bragg reflections with rotational point symmetries that are inconsistent with periodic translational order [1]. The resolution limited Bragg peaks observed for the best ordered icosahedral quasicrystals demonstrate that the atomic order is truly long-range, albeit aperiodic. In the 30 years since their discovery there have been significant advances in our knowledge of the atomic scale structure [2, 3], but many questions regarding the consequences of aperiodicity on physical properties, such as electronic and magnetic properties, remain open. Perhaps one of the most interesting questions regarding magnetism in quasicrystals, as yet unanswered, is whether long-range antiferromagnetic (AFM) order can be sustained in real quasicrystalline systems. A large number of theoretical studies have focused on the possibility of non-trivial ordering of localized magnetic moments on quasilattices [4–14], and generally answered affirmatively. For example, in early work, Lifshitz demonstrated that symmetry considerations admit simple AFM order for primitive and body-centered icosahedral quasilattices, but not for face-centered icosahedral (FCI) quasicrystals [7, 8, 15, 16]. Nevertheless, to date, no ‘quasiantiferromagnets’ have been discovered. That is not to say that the low-temperature behavior of the known magnetic quasicrystals is uninteresting. Quite the contrary, they continue to offer new insights regarding the roles of topological order and frustration, as well as the microscopic nature of complex spin interactions in magnetic systems.

Studies of magnetism in the first generation of Al-based quasicrystals that contained 3d transition metals, such as Mn and Fe, were undertaken very soon after the discovery of icosahedral Al-Mn [17–19], and have been discussed in reviews by OʼHandley, Dunlap and McHenry in 1990 [20] and, more recently, by Hippert and Préjean in 2008 [21]. However, investigations of the impact of quasiperiodicity on local moment interactions in the Al-base quasicrystals were hampered by several issues. First, the magnetic moment in the Al-*TM* (*TM* = 3d transition metal) cannot be regarded as truly localized since it originates from the itinerant 3d electrons. Secondly, the number of *TM* sites as well as their local environments and site symmetries were not well established. The essential conclusions from years of study are that (i) the moment formation in these systems is highly dependent upon the local environment of the *TM* and varied strongly with the alloy composition; (ii) only a small fraction of the Mn or Fe ions carry a moment (at most a few percent); and (iii) the moments that do form appear to carry a large spin value and are distributed randomly on the quasilattice. It is not surprising then, that long-range magnetic order is not realized in these quasicrystals and the low-temperature ground states can best be characterized as site-disordered canonical spin-glasses (SG) such as noble metals doped by magnetic ‘impurities’ [22].

The discovery of the *R*-Mg-Zn [23–26] and *R*-Mg-Cd [27, 28] (*R* = rare earth) quasicrystals stimulated new investigations of the magnetic properties of quasicrystals and reinvigorated the search for long-range magnetic order in the icosahedral phase. These systems offer the distinct advantage of well-localized magnetic moments, originating from the 4*f* electrons of the *R* ions, that interact via the indirect Ruderman–Kittel–Kasuya–Yosida exchange [29–31]. Therefore, the question of moment formation versus moment interactions on the quasilattice could effectively be resolved. Furthermore, very large single grains of the *R*-Mg-Zn quasicrystals can be obtained via flux-growth methods [32]. Two decades of experimental work on the *R*-Mg-Zn and *R*-Mg-Cd icosahedral quasicrystals have provided a wealth of information regarding the character of the low-temperature SG-like state in these quasicrystals and have also raised many issues that have yet to be resolved. Previous comprehensive reviews of this work have been presented by Fukamichi in 1999 [33], Sato in 2005 [34], and Dolinšek and Jagličić in 2012 [35].

The discovery of stable binary icosahedral quasicrystals in Yb-Cd and Yb-Ca [36, 37] and the elucidation of the structure of these ‘Tsai-type’ quasicrystals [38] mark important points in the timeline of magnetic property investigations even though, at ambient pressure, they do not feature local moments. The substitution of Zn for Cd, Sc for Yb, and the addition of elements such as Mg, Mn, Fe, Co and Ni resulted in several new Sc-*TM*-Zn ternary quasicrystals [39, 40]. The bulk magnetic properties of the Zn-based icosahedral quasicrystals have been investigated, as have some of their periodic 1/1 approximants based on the 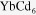 and 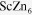 structures, thus allowing a comparison between the SG-like ground states of the quasicrystalline and closely related periodic phases. Similarly, through the substitution of Ag and In, or Au and Al, for Cd, *R*-Ag-In [41, 42] and Yb-Au-Al [43] icosahedral quasicrystals have been realized and their magnetic properties studied in comparison to those of their respective periodic approximant phases. Whereas the Gd-Ag-In quasicrystal and approximant both exhibit SG-like behavior [44], the Yb-Au-Al quasicrystal manifests novel quantum critical behavior at low temperature [45].

Most recently, the discovery of binary icosahedral quasicrystals in the *R*-Cd (*R* = Gd-Tm, Y) system [46] offers yet another opportunity for the investigation of magnetism in quasicrystals and attaining a deeper understanding of the nature of magnetic interactions in aperiodic systems. The new *R*-Cd quasicrystals may play a key role as the simplest magnetic quasicrystal system, offering non-magnetic (*R* = Y), Heisenberg-like (*R* = Gd), and non-Heisenberg (e.g., crystal-electric field (CEF) split Tb to Tm members), as well as the structural and compositional simplicity of a binary phase with well-defined *R* sites. Perhaps most importantly, they are closely related to the much studied *R*Cd_6_ cubic approximants which do, in fact, manifest long-range magnetic order at low temperatures [47–51]. Given the chemical simplicity associated with a binary compound, the matched sets of i-*R*-Cd and *R*Cd_6_ form model systems that should allow us to determine, refine and test our understanding of the key features and properties associated with quasicrystalline structure and magnetism.

## 2. Clusters in icosahedral quasicrystals and approximants featuring rare-earths

Before proceeding with a discussion of bulk and microscopic measurements of magnetism in *R*-containing quasicrystals, it will be useful to briefly describe some of the structural elements that come into play. Two primary structural types are discussed in this review. The first *R*-containing quasicrystals, *R*-Mg-Zn, were reported by Luo *et al* [23] and later established as FCI structures [24–26]. Primitive, or P-type, icosahedral quasicrystals in the *R*-Mg-Zn have also been synthesized [52] and studied [53]. Both types of *R*-Mg-Zn quasicrystals feature Bergman-type clusters [54] comprised of concentric shells around a central void: a 12-atom inner icosahedron; a 20-atom pentagonal dodecahedron; a 12-atom outer icosahedron; and finally a 60-atom soccer ball. The *R* ions are believed to occupy the vertices of the pentagonal dodecahedron, together with Mg-atoms, to form an edge-sharing linked network of *R*-containing clusters in the quasicrystal [34].

Quasicrystal approximants are periodic crystals with compositions and unit-cell atomic decorations (for example, atomic clusters) that are closely related to their respective quasicrystalline phases. They provide an important link between periodic and aperiodic crystals both in terms of assisting in structural studies and comparative studies of the physical properties to provide a means of identifying anomalous behavior that is derived from the absence of periodicity in quasicrystals. For example, the 1/1 and 2/1 rational approximants of the 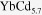 quasicrystal were key to the successful refinement of the quasicrystalline structure [38]. Rational approximants refer to periodic crystals that can be derived from rational projections of the six-dimensional hypercubic lattice describing the quasicrystal [55]. For the *R*-Mg-Zn quasicrystals no simple rational approximants have been found although the hexagonal phase of Ho-Mg-Zn, close in composition to the FCI phase, has been used in neutron scattering studies for a comparison of the inelastic excitation spectra. A 2003 by review Sterzel *et al* describes the preparation of *R*-Mg-Zn quasicrystals and related compounds [56].

**Figure 1.**
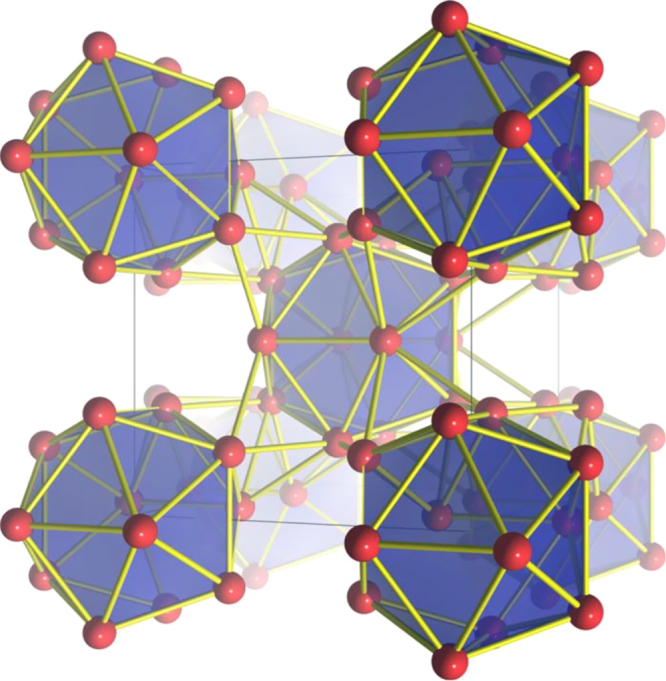
*R* sites in the unit cell of the *R*Cd_6_ cubic approximant to the Tsai-type icosahedral quasicrystals. After [47].

The second major class of magnetic quasicrystals are P-type icosahedral phases based on the Tsai-type clusters found in the stable binary quasicrystal 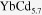 [36]. The Tsai-type clusters in 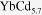 are comprised of four successive shells surrounding a tetrahedron of four Cd atoms: a dodecahedron composed of 20 Cd atoms; an icosahedron of 12 Yb atoms, an icosidodecahedron of 30 Cd atoms; and an outermost shell described as a defect rhombic triacontahedron of 60 Cd atoms. The 1/1 cubic approximant, 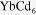 , and the corresponding *R*Cd_6_ 1/1 approximants to the *R*-Cd, *R*-Mg-Cd, Sc-Fe-Zn, *R*-Ag-In and Yb-Au-Al ternary quasicrystals discussed here, may be described, at ambient temperature, as a body-centered cubic packing of interpenetrating Tsai-type clusters, which features an icosahedron of 12 *R* atoms comprising the third shell of the cluster. These clusters are linked along the cubic axes by sharing a face, and interpenetrate neighboring clusters along the body diagonal [57]. The network of face-sharing *R*-icosahedra is illustrated in figure 1. One simplification offered by *R*Cd_6_ is that the *R* ions are found at a single crystallographic site in the lattice. For 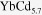 , at least, these same clusters have been shown to comprise the backbone of the structure of the icosahedral phase with the same type of linkages [38]. At temperatures between 100 and 200 K *R*Cd_6_ undergoes a cubic-to-monoclinic distortion that has been studied extensively and reviewed recently by Tamura [58].

## 3. Magnetism in ternary icosahedral quasicrystals

### 3.1. *R*-Mg-Zn and *R*-Mg-Cd

**Figure 2.**
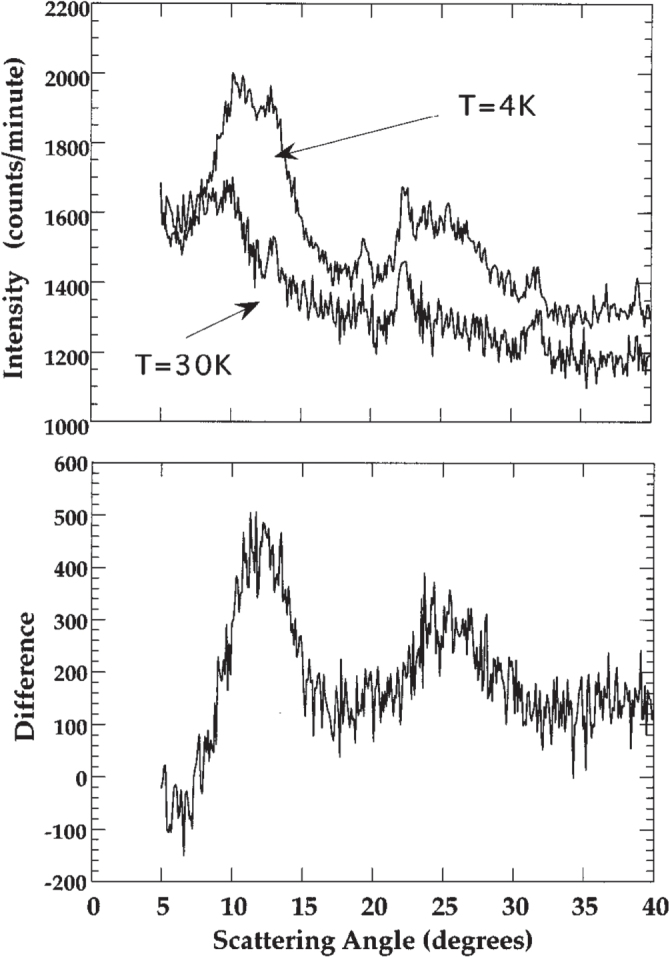
Neutron diffraction from icosahedral Tb-Mg-Zn. The top panel displays the neutron-diffraction data taken at 4 and 30 K on crushed single grains. The data in the bottom panel results from a subtraction (I(4 K)-I(30 K)) of the data from the top panel, emphasizing the onset of short-range magnetic ordering, and the absence of any sharp magnetic component, at low temperature. After [66].

Early neutron powder diffraction measurements on icosahedral 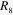 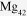 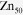 noted the appearance of sharp magnetic Bragg peaks, suggesting the onset of AFM order in these quasicrystals, along with broad diffuse scattering below the reported 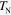 of 5.8, 3.8, 2 and 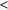
1.5 K, respectively, for *R* = Tb, Dy, Ho and Er [59]. However, no corresponding sharp anomalies were observed in magnetic susceptibility or specific heat measurements [60–63] and spontaneous spin precession was not observed in *μ*SR measurements below 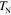 [64, 65]. Later neutron diffraction measurements using crushed single grains of 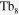 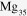 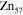 [66] and powders and single grains of icosahedral Ho-Mg-Zn [67–69] failed to reproduce the sharp magnetic Bragg peaks (see figure 2). It was concluded that the sharp magnetic Bragg peaks reported earlier were likely from a second phase contained within the as-cast 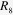 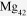 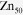 samples.

In fact, all of the known *R*-Mg-Zn and *R*-Mg-Cd icosahedral quasicrystals with moment bearing elements exhibit spin-glass-like behavior at low temperature rather than long-range magnetic order. The true microscopic nature of the frozen state, however, remains a matter of discussion largely focused on identifying the degree of freedom that is frozen at low temperature. Are magnetic quasicrystals best characterized in terms of a canonical site-disordered SG, or site-ordered geometrically frustrated systems [70–72] that can range from SG-like ground states to superparamagnetism arising from non-interacting clusters of strongly coupled moments [35]? At a minimum, the low-temperature magnetic behavior of these quasicrystals appears to be unusual.

**Figure 3.**
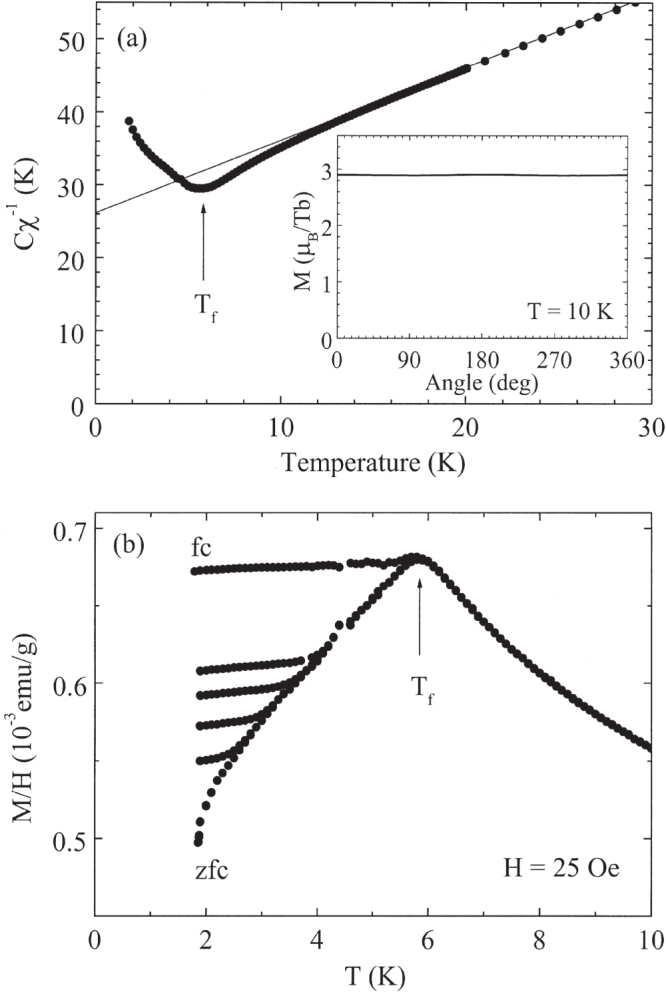
(a) The inverse susceptibility of icosahedral Tb-Mg-Zn, measured in an applied field of 1 kOe. The line depicts the extrapolation of the high-temperature fit to the Curie–Weiss law. The inset shows the angular dependence of the dc magnetization of Tb-Mg-Zn at 10 K (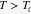) (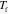 = spin freezing temperature) and in an applied field of 55 kOe. (b) The temperature dependence of the dc magnetization of Tb-Mg-Zn in an applied field of 25 Oe, following an initial zero-field-cool or field-cool (labeled in figure). Additional data taken following a field-cool from temperatures less than 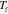 (from 2.5, 3.0, 3.5 and 4.0 K) are also shown. After [75].

Bulk property measurements on the *R*-Mg-Zn and *R*-Mg-Cd quasicrystals have been pursued by many groups around the world [61, 62, 73–79] and have been discussed in some detail by Sato [34]. As illustrated in figure 3(a) for Tb-Mg-Zn, at high temperatures, the inverse magnetic susceptibilities of the *R*-Mg-Zn icosahedral quasicrystals are isotropic and closely follow the Curie–Weiss law with effective moments that are consistent with the free 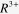 values (see table 1). The Weiss temperatures derived from fits using the Curie–Weiss law are negative, indicating primarily AFM interactions between the *R* ions. At low temperature, a cusp-like feature associated with a spin-freezing temperature (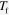) is observed in the zero-field-cooled (ZFC) magnetic susceptibility below which significant irreversibility is found for ZFC and field-cooled (FC) magnetization measurements (figure 3(b)). Furthermore, a cusp in the real part of the ac-magnetic susceptibility (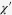) measurements was observed, which shifts in temperature as the applied field is varied [73]. Although these features point to canonical spin-glass freezing below 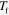 [22], they may also be attributed to site-ordered geometrically frustrated spin systems [35] and superparamagnetic clusters below the blocking temperature [80]. The ratio 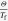 for all of the *R*-Mg-Zn quasicrystals is approximately 4, classifying them as moderately frustrated magnetic systems.

**Table 1.** Magnetic properties of the ternary *R*-Mg-Zn and *R*-Mg-Cd icosahedral quasicrystals. Specific compositions are provided when known. 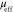 is the measured effective moment per *R*-ion, 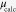 is the calculated free-ion value, 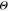 is the measured Weiss temperature, and 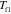 and 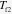 are the spin freezing temperatures (if observed).

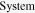	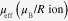	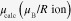	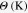	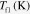	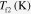	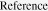
Gd-Mg-Cd	7.9	7.94	−37	13.0	4.8	[77]
Gd-Mg-Cd	7.24		−37.8	4.3	—	[79]
Tb_8_ Mg_42_ Zn_50_	10.05	9.72	−26	5.8	—	[62]
Tb_9_ Mg_34_ Zn_57_	9.91		−26.3	5.8	—	[73]
Tb-Mg-Cd	10.03		−23	12.5	5.6	[77]
Tb-Mg-Cd	9.74		−24.5	5.9	—	[79]
Dy_8_Mg_42_Zn_50_	9.78	10.63	−17.2	3.8	—	[62]
Dy-Mg-Zn	10.5		−14.8	3.6	—	[73]
Dy-Mg-Cd	10.67		−14	7.4	3.8	[77]
Dy-Mg-Cd	10.59		−18.4	3.2	—	[79]
Ho_8_ Mg_42_Zn_50_	9.79	10.60	−10	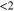	—	[62]
Ho-Mg-Zn	10.4		−7.8	1.95	—	[73]
Ho-Mg-Cd	10.42		−7	12.5	5.0	[77]
Er_8_ Mg_42_ Zn_50_	9.59	9.59	−6.3	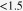	—	[62]
Er-Mg-Zn	9.49		−5.1	1.30	—	[73]
Er-Mg-Cd	9.71		−6	4.4	—	[77]
Tm-Mg-Cd	7.08	7.57	-2	—	—	[77]

For both Tb-Mg-Zn and Ho-Mg-Zn, Fisher *et al* [73] observed a sharp peak in the third-order susceptibility, 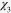, which is consistent with a canonical site-disordered spin-glass freezing scenario. Furthermore, the relaxation of the magnetization below 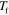 was measured and could be fit to a stretched exponential function, again consistent with spin-glass behavior [22]. However, these features are also found for site-ordered geometrically frustrated systems [34, 35]. Later measurements of the thermoremnant dc-magnetization (TRM) decay by Dolinšek *et al* found a linear increase with the cooling magnetic field below 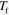 which, they note, is incompatible with canonical spin-glass behavior since it indicates a single global free-energy minimum characteristic of the blocking of spins in finite-sized non-interacting clusters [78].

**Figure 4.**
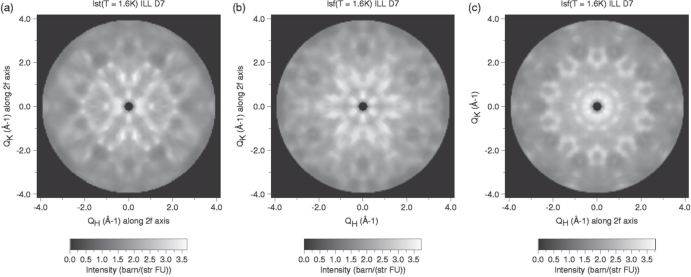
Magnetic-scattering intensity maps at *T* = 1.6 K for the (a) twofold, (b) threefold, and (c) fivefold planes obtained from the spin-flip scattering of polarized neutrons from icosahedral Ho-Mg-Zn. After [68].

Both elastic and inelastic neutron scattering measurements on single grains of *R*-Mg-Zn icosahedral quasicrystals have been performed with some interesting results related to the discussion above. In particular, unpolarized and polarized neutron diffraction measurements on a single grain of icosahedral Ho-Mg-Zn clearly showed the presence of anisotropic magnetic diffuse scattering with icosahedral symmetry at low temperature shown in figure 4 [67, 68]. The diffuse scattering first appears at temperatures well above the 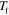 measured in bulk magnetization studies. This difference likely arises as a result of the different time scales associated with bulk property and neutron scattering measurements [81]. The full-width-at-half maximum (FWHM) of the most prominent diffuse peak was used to establish a magnetic correlation length, *ξ* ∼20 Å, for the regions that give rise to the magnetic scattering. This is comparable to the size of dodecagonal *R* clusters, within the Bergman-type clusters, that comprise an edge-sharing network in the quasicrystalline structure [82]. Therefore, Sato has proposed a model for the magnetic diffuse scattering that originates from strong correlations between moments on the same dodecagonal cluster [34]. As he notes, however, there is no clear reason why moments on adjacent clusters of the edge-sharing network should not interact as well, and this question remains unresolved to date. Nevertheless, the magnetic diffuse scattering pattern could be reasonably reproduced using a classical Heisenberg Hamiltonian with only second-neighbor interactions. This, of course, ignores the single-ion anisotropy that arises from crystal field effects. The resulting ground-state spin structure is non-collinear and continuously degenerate.

**Figure 5.**
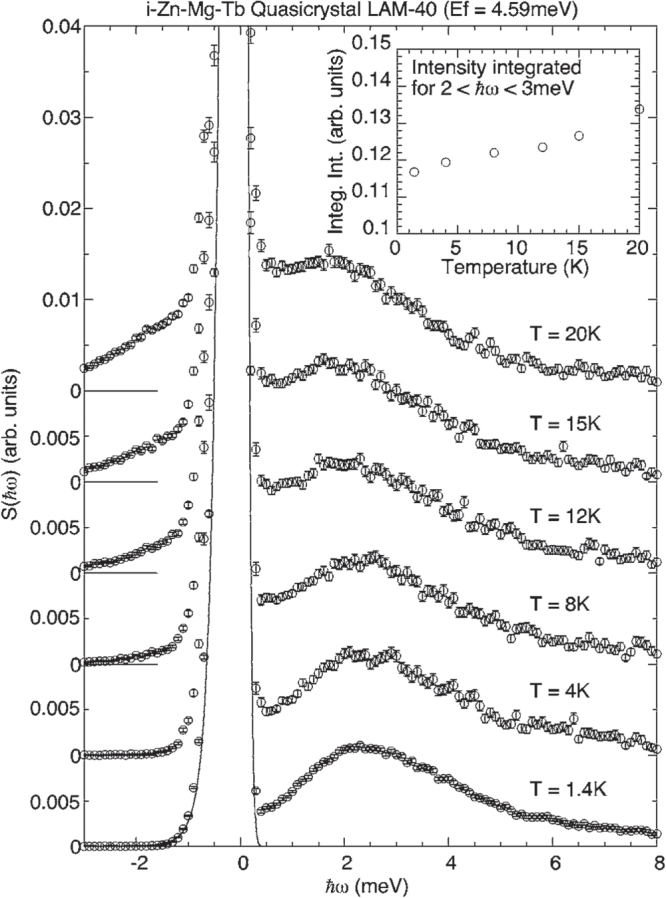
*Q*-integrated scattering function, S(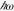), for icosahedral Tb-Mg-Zn at several temperatures. The intensities observed at several angles covering 2 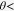 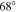 (*Q* 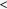 1.7 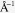 at the elastic position) were summed to increase statistics. The solid line represents the vanadium standard spectrum as the instrumental resolution function. Inset: temperature dependence of the integrated intensity for 2 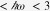 meV. After [83].

Powder and single-grain inelastic neutron scattering measurements have been performed on icosahedral Tb-Mg-Zn [83] with several interesting results. As shown in figure 5, a single broad, non-dispersive inelastic peak was observed at an energy transfer of 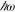 = 2.5 meV that persists down to the base temperature (1.4 K) of the measurement. The intensity of the peak, over a relatively narrow temperature range (∼1–15 K), increases slightly, rather than decreases, and a detectable anisotropy in the intensity distribution of the excitation that resembles the static magnetic diffuse scattering was observed. These features led the authors to exclude its origin as crystalline electric field (CEF) excitation and, instead, attribute the inelastic peak to a localized spin excitation analogous to the boson peak seen in topological glasses [84], corresponding to collective fluctuations of spins on a cluster. A strong quasielastic signal appears in their high-resolution scattering data above 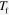 = 5.8 K which vanishes at 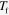. The coexistence of the inelastic and quasielastic peaks for *T* 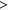 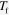 suggests that the dynamic short-range correlations above 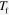 in these clusters freeze at 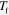. So, in this picture, the degree of freedom that freezes at 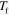 are the fluctuations associated with the entire cluster.

**Figure 6.**
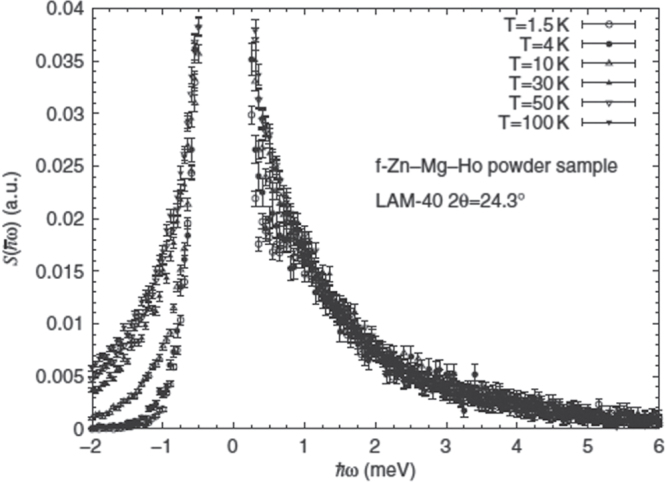
Neutron inelastic scattering spectra of a Ho-Mg-Zn powder sample observed at several temperatures between 1.5 K and 100 K. After [85].

Very different behavior of the magnetic excitations have been observed from inelastic neutron scattering measurements on Ho-Mg-Zn [85] as shown in figure 6. Rather than a peak at finite energy transfer, as found for Tb-Mg-Zn, only a broad quasielastic signal centered at 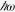 = 0 was observed. The absence of a collective mode in the inelastic scattering and negligible Q-dependence, aside from the magnetic form factor, implies relaxational single-site (rather than cluster) spin dynamics in the Ho-Mg-Zn quasicrystal. Perhaps most intriguing, however, is their observation of temperature independent scattering in the energy-loss spectrum (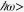 0). Their analysis of the data suggests that the spin fluctuations do not scale with any characteristic temperature (e.g. 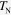 for antiferromagnets, or 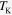 for Kondo systems) but, rather, exhibit 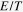 scaling seen previously for heavy fermion systems with non-Fermi-liquid behavior [86]. Neither the origin of this scaling, nor the reason for the differences in the inelastic scattering for Ho-Mg-Zn and Tb-Mg-Zn are understood at this point. Finally, in the same work, the authors also measured the inelastic neutron scattering from hexagonal 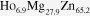 [87]. In contrast to what was found for the Ho-Mg-Zn and Tb-Mg-Zn quasicrystals, well-defined CEF excitations were observed at low temperature in the inelastic scattering. Inasmuch as the crystalline phase features only two *R* sites, this suggests that the absence of distinct CEF excitations in the quasicrystalline phases may, in fact, arise from a multiplicity of sites with different local environments.

Whereas the *R*-Mg-Zn quasicrystals are classified as face-centered icosahedral structures that feature Bergman-type clusters, the *R*-Mg-Cd quasicrystals are primitive icosahedral phases that contain Tsai-type clusters. For *R*-Mg-Zn, approximately 2/3 of the *R* ions are located on the network of edge-sharing dodecahedra. For *R*-Mg-Cd approximately 70% of the *R* ions are located at the vertices of an icosahedron which comprises the third shell of the Tsai-type cluster. These *R*-decorated icosahedra form a network connected along the triangular faces of the icosahedra. Despite the global differences in structure and cluster-type, the magnetic behavior of *R*-Mg-Zn and *R*-Mg-Cd are remarkably similar in terms of their values for 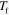 and 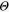 (see table 1). However, the SG-like transition in *R*-Mg-Cd quasicrystals seems to be even more complex.

**Figure 7.**
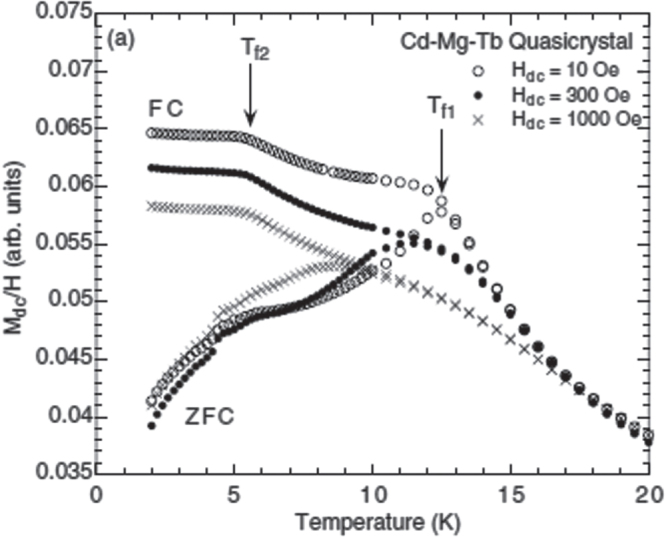
Magnetization curves of the polycrystalline sample of the Tb-Mg-Cd quasicrystal measured under an applied fields of 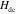 = 10, 300 and 1000 Oe for field cooled (FC) and zero-field-cooled (ZFC) histories. After [77].

**Figure 8.**
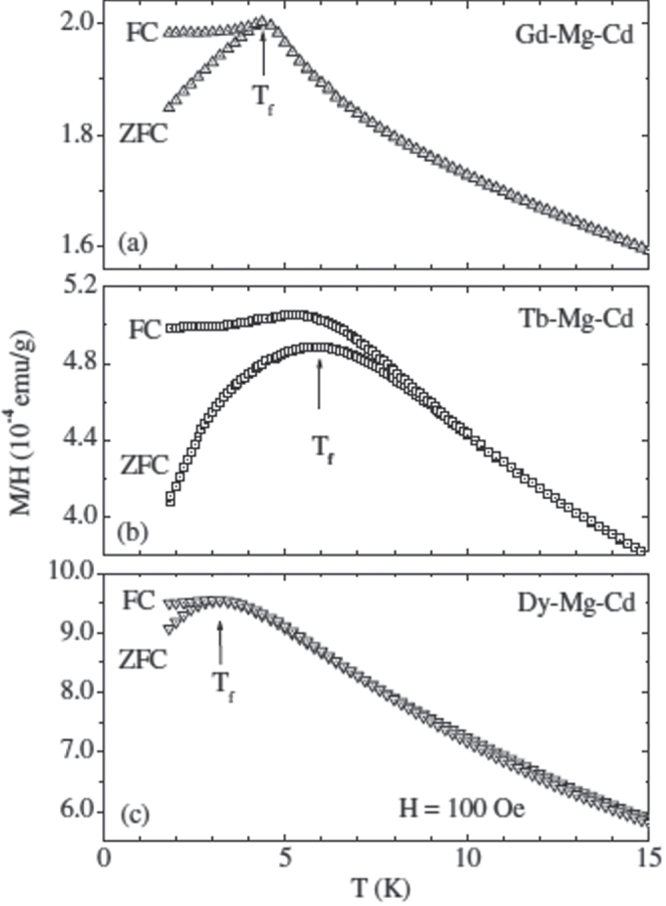
Magnetization curves of single grains of R-Mg-Cd measured under 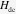 = 100 Oe showing both FC and ZFC behaviors: (a) Gd-Mg-Cd; (b) Tb-Mg-Cd; (c) Dy-Mg-Cd. After [79].

At high temperature, like the *R*-Mg-Zn icosahedral quasicrystals, the inverse magnetic susceptibilities of the *R*-Mg-Cd icosahedral quasicrystals are isotropic and closely follow the Curie–Weiss law with effective moments that are consistent with the free 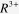 values. At low temperature, features associated with spin-glass-like freezing are again observed [77, 79] but, as shown in figure 7, measurements on polycrystalline samples exhibit two anomalies, corresponding to two possible freezing events, labeled 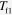 and 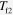 [77]. Two anomalies are also observed in the temperature dependence of 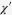 in ac-susceptibility measurements. Figure 8 displays dc-susceptibility measurements on single grain samples by Sebastian *et al* [79], which evidence only a single broad feature that peaks at ∼ 6 K, for 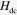 = 100 Oe, which they label as 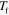. The broadened cusp in these magnetization measurements may indicate that there is a range of freezing temperatures for this system. However, both the polycrystalline and single grain data for the Tb-Mg-Cd quasicrystal show that the maximum in the FC data occurs below the onset of irreversibility in this system, which is unusual.

In an attempt to shed some light on the microscopic origin of these two temperature scales, neutron diffraction measurements on ^114^ Cd-isotope substituted polycrystalline samples of Tb-Mg-Cd were measured at temperatures between 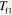 and 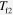 and below 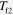 [88] with the results that (i) the magnetic diffuse scattering characteristic of short-range magnetic correlations similar to those observed for the *R*-Mg-Zn icosahedral quasicrystals developed upon cooling the sample below 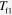; and (ii) no change in the magnetic diffuse scattering was evident at 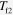. The odd magnetization curve of Tb-Mg-Cd at low temperature remains an unresolved issue.

### 3.2. Sc-Fe-Zn

The prototype of the Sc-*TM*-Zn quasicrystals (*TM* = Mn, Fe, Co, Ni) is 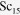 Mg_5_ 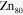 [39] which shows diamagnetism and Pauli paramagnetism. A 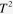-dependence of the magnetic susceptibility was observed for both the quasicrystal and the closely related ScZn_6_ 1/1 cubic approximant, consistent with the presence of a pseudogap in the density of states at the Fermi energy [89]. With the introduction of 3d *TM* elements, however, Curie–Weiss-like paramagnetism is recovered with effective moments substantially larger than what was previously observed for the Al-based quasicrystals [90–92]. For the Sc-*TM*-Zn quasicrystals it is generally presumed that the *TM* substitutions occupy the Zn-sites in the structure. This differentiates this system from others derived from the binary 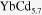 structure where the magnetically active ion is associated with the icosahedron of *R* ions that comprise the third shell of the Tsai-type clusters.

**Figure 9.**
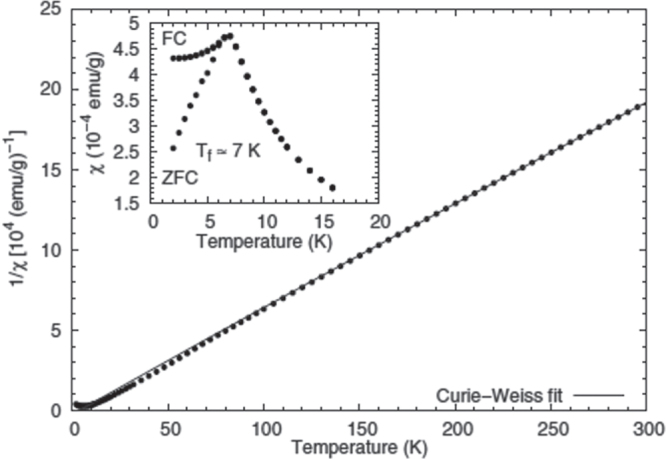
Inverse susceptibility of quasicrystalline Sc_16_Fe_7_Zn_77_ obtained from the temperature dependence of the magnetization measured under a dc external field of 600 Oe. The solid line represents a Curie–Weiss fit in the temperature range 200 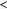 *T* 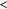 300 K. Inset: expanded view of the low temperature region (*T* 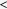 20 K) observed under the dc field of 50 Oe. The field-cooled (FC) and zero-field-cooled (ZFC) susceptibilities are shown. After [93].

The Sc-Fe-Zn system is of particular interest since the effective Fe moment for icosahedral phase of 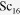 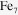 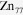 was originally estimated to be approximately 5.3 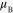/Fe, intermediate between the 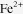 and 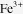 free ion values, with a positive 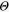 = 6.5 K from a fit of the magnetic susceptibility to a Curie–Weiss law, indicating primarily ferromagnetic interactions [91]. A cusp in the dc susceptibility at 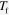 = 7.2 K and splitting between the FC and ZFC curves again signals a SG-like transition. Later measurements on icosahedral quasicrystals with the same starting compositions found somewhat different values for the effective moment and 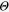 of 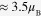/Fe and ≈ 11 K [92], or 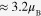/Fe and ≈ 3 K [93], but very similar values for 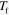 (figure 9). The source of discrepancies in the measured values for the effective moment has not been identified although it was speculated that even small changes in composition may strongly affect the moment value as was noted for the Al-based magnetic quasicrystals [21]. Similarly, the wide range of values for 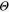 was taken as an indication of the presence of both ferromagnetic and AFM interactions of similar magnitude that nearly cancel in the total spin–spin interactions in the system. Again, small changes in composition or disorder would then affect this balance strongly [93].

A comprehensive set of structural and bulk property measurements on powder samples of the Sc-Fe-Zn quasicrystal was reported by Al-Qadi *et al*, including x-ray diffraction, dc and ac susceptibility, TRM decay and ^57^Fe Mössbauer spectroscopy [92]. The powder diffraction peak widths were quite narrow indicating a high degree of structural order in these samples and, in particular, there was no visible dependence of the peak widths on the phason momentum, 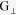 [94], indicating the absence of any significant frozen in phason strain [95]. Both the dc and ac susceptibility exhibited features at 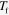 consistent with SG freezing. However, once again, the magnitude of the TRM was observed to increase with the cooling field, rather than decrease as expected for a canonical SG. Their Mössbauer spectroscopy measurements showed a bimodal distribution of the quadrupole splitting, suggesting the presence of two classes of Fe sites in the quasicrystal. Other ^57^Fe Mössbauer spectroscopy measurements on both the 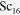 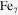 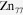 quasicrystal and the 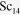 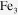 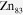 periodic 1/1 approximant phase, however, found significant differences in their Fe local environment evidenced by a broad single-peaked distribution for the quasicrystal (similar to previous measurements on Al-based quasicrystals) and a two symmetric doublets for the approximant phase [96].

Elastic and inelastic neutron scattering measurements have been performed on powders of the 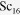 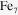 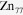 icosahedral phase [93]. It was found that the magnetic scattering exhibits not only a diffuse component associated with the onset of static short-range correlations at low temperature (but, again, well above 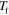), as observed previously in the *R*-Mg-Zn and *R*-Mg-Cd quasicrystals, but also a *Q*-independent quasielastic scattering component characteristic of single-site relaxational spin dynamics. A quantitative comparison of the normalized diffuse scattering between the Sc-Fe-Zn and Tb-Mg-Zn quasicrystals noted that the amplitude of the oscillations with momentum transfer, *Q*, were quite comparable, leading the authors to conclude that the intersite correlations were very similar. This is a bit surprising since (i) the *R*-Mg-Zn and Sc-Fe-Zn quasicrystals belong to different structure types (a FCI Bergman-cluster phase versus the primitive icosahedral Tsai-cluster phase) and (ii) the *R* and *TM* atoms occupy very different sites within either structure. Lorentzian fits to the quasielastic scattering as a function of temperature yielded an energy scale for the fluctuations, 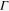, which increased in an Arrhenius fashion above 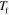, but saturated to a finite value below 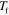, leading the authors to speculate that there was significant on-site scattering of the 3*d* moments by conduction electrons.

### 3.3. *R*-Ag-In

The substitutions of other *R* ions for Yb, and Ag and In for Cd in Yb-Cd led to the discovery of both quasicrystalline [41, 42] and periodic approximant phases [97, 98] in the *R*-Ag-In system, and both phases have been studied using a variety of bulk and microscopic probes. The magnetic properties of the 1/1 cubic approximant phases were investigated for the full series of *R* ions [99]. For *R* = Nd, Eu, Gd, Tb, Dy, Ho, Er and Tm, the dc magnetic susceptibilities at high temperature (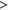 100 K) exhibit Curie–Weiss behavior and the effective moments are consistent with the free ion values for 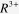 (or 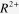 for Eu). The Weiss temperatures derived from the fit ranged from –55 K for *R* = Gd to –4 K for *R* = Tm, and followed the de Gennes factor. For Sm and Yb, the magnetic susceptibility was quite small suggesting that they are both found in the non-magnetic divalent state and, for Ce and Pr, the magnetic susceptibility was nonlinear pointing to strong CEF or intermediate valence effects.

What is, perhaps, most interesting about this series is that both the quasicrystals and approximants manifest SG-like freezing at low temperature. For the *R*-Ag-In approximants, the low temperature magnetic susceptibility measurements revealed a splitting between the FC and ZFC susceptibilities for *R* = Eu, Gd, Tb and Dy with 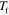 largest for Tb (3.7 K). Taking the ratio of 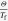 again as a measure of magnetic frustration in the system, they found values ranging from 17 for *R* = Gd to 7 for *R* = Tm classifying the approximants as highly frustrated magnetic systems [99].

Magnetic susceptibility measurements of quasicrystalline Gd-Ag-In at high temperature found an effective Gd moment consistent with the free ion value and a value for 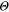 = –37 K [100], somewhat smaller than found for the corresponding approximant. 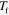 for the quasicrystal, determined from the temperature of the cusp in the dc magnetic susceptibility, was found to be 4.25 K, somewhat higher than the Gd-Ag-In cubic approximant. These comparisons suggest a higher degree of magnetic frustration in the approximant phase. ac magnetization measurements on the approximant and quasicrystal further found a double-peaked structure for the in-phase susceptibility, 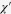, for the approximant phase (similar to what was found in some measurements on quasicrystalline *R*-Mg-Cd [77]), whereas the quasicrystal featured only a single anomaly [44]. This was interpreted in terms of a two-step freezing process in the approximant where the moments develop short-range correlations at 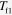 ≈ 3.6 K but continue to fluctuate at low frequencies, and then freeze at 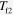. However, ^155^Gd Mössbauer spectroscopy of the icosahedral phase [100] and the approximant [44] above 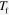 found close values for the effective quadrupole splitting parameter, 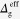, suggesting that the Gd local environments were quite similar in the quasicrystal and approximant phases. Furthermore, the fit values for the linewidths for both the approximant and icosahedral phase indicate a multiplicity of Gd sites that likely arises from a random distribution of Ag and In on the Cd sites in both the approximant and quasicrystal phases.

Elastic and inelastic neutron scattering measurements on powders of the Tb-Ag-In 1/1 cubic approximant have been reported [99] and, similar to previous measurements on the magnetic quasicrystals, significant structure in the magnetic diffuse scattering, signifying magnetic short-range order, was observed to develop at 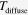 ∼60 K, well above 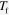 = 3.7 K. Inelastic scattering measurements showed a strong decrease in the energy width of the quasielastic scattering (corresponding to slowing of the spin fluctuations) below approximately 100 K, and the emergence of an inelastic peak at 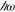 = 4 meV below 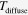. Indeed, the inelastic scattering from the Tb-Ag-In approximant seems quite similar to that from the Tb-Mg-Zn icosahedral quasicrystal where the inelastic peak was associated with correlated fluctuations among the moments on clusters below 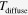 which then freeze at 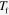.

### 3.4. Yb-Au-Al

The Yb-Au-Al icosahedral phase and its 1/1 cubic approximant [43] are also closely related to the 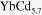 quasicrystal and the 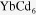 approximant, and physical property measurements of these systems have revealed some fascinating new magnetic behavior [45, 101]. Magnetic susceptibility measurements at high temperature found very similar behavior for both the quasicrystal and approximant and fits using the Curie–Weiss law to the data yielded effective moments of 3.91 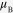/Yb(3.96 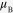/Yb) and Weiss temperatures of –153 K(–117 K) for the quasicrystal (approximant). This is in striking contrast to the absence of a local moment for 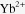 in the 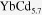 quasicrystal and 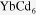 approximant at ambient pressure, although intermediate valence behavior has been observed in Yb-Cd and Yb-Mg-Cd quasicrystals under applied pressure [102–104]. The effective moments for the Yb-Au-Al systems are intermediate between 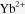 and 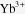 , classifying them as mixed valent as confirmed by x-ray absorption near edge structure measurements [101]. In addition, the Weiss temperatures for both are significantly higher than previously studied magnetic quasicrystals and approximants indicating strong AFM interactions.

**Figure 10.**
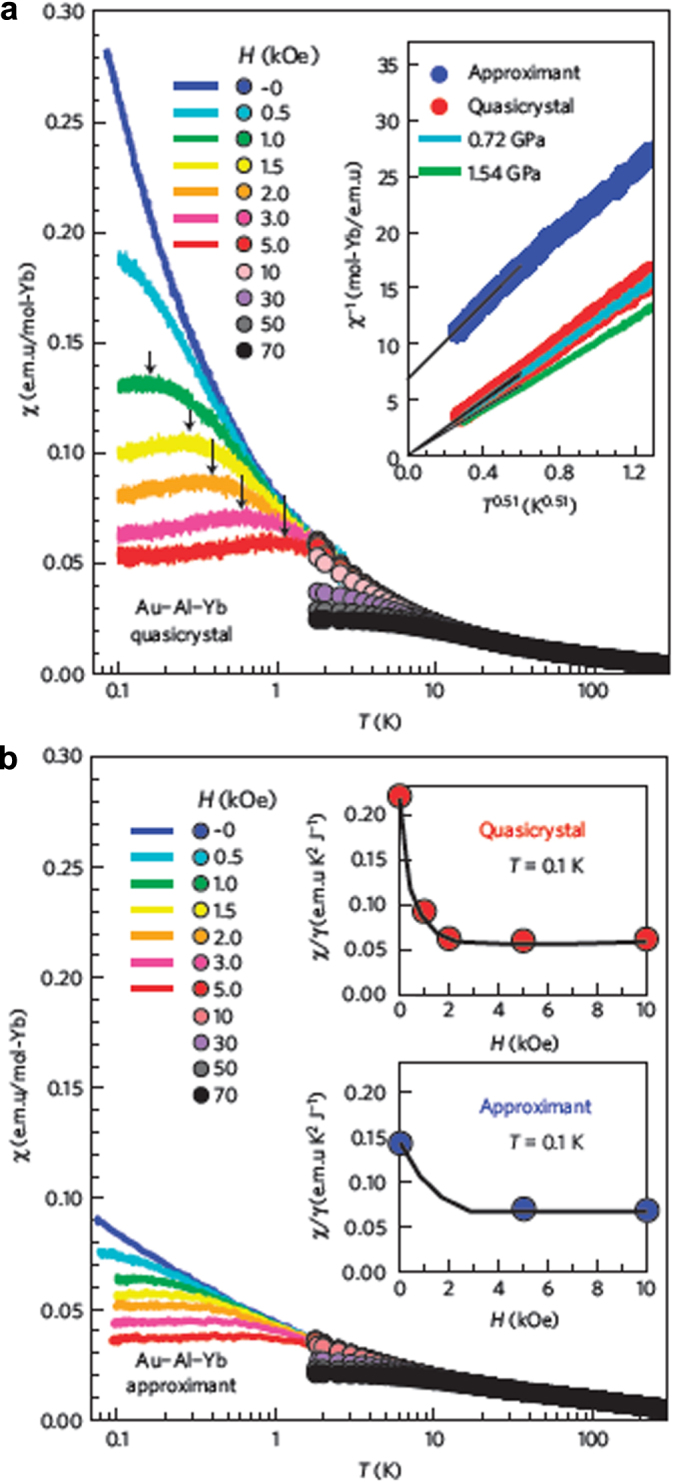
Temperature dependence of the magnetic susceptibility of the Yb-Au-Al quasicrystal and approximant. (a) ac and dc magnetic susceptibility of the quasicrystal measured in a temperature range of 0.08 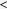 *T* 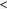 3.0 K (denoted by the lines) and 1.8 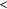 *T* 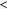 300 K (circles), respectively. Magnetic fields are described in the figure. The abscissa is plotted on a logarithmic scale. Inset shows the inverse susceptibility 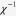 versus 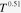 of the approximant (blue circles) and the quasicrystal (red circles) at ambient pressure, and of the quasicrystal at pressures of 0.72 GPa and 1.54 GPa. The black lines are a linear extrapolation to *T* = 0. (b) Magnetic susceptibility of the approximant measured in the same condition as in panel (a). Insets show the field dependence of the ratio 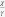 at *T* = 0.1 K for the quasicrystal and the approximant, respectively, where 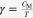. After [45].

Although the high temperature behaviors of the quasicrystal and approximant are quite similar, measurements by Deguchi *et al* demonstrated a dramatic difference at low temperature (figure 10) [45]. Neither the quasicrystal nor the approximant evidence any SG-like freezing at low temperature but, whereas the approximant phase exhibits conventional Fermi-liquid behavior at low temperature (*ρ* ∼ 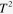 and neither 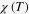 nor 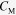/*T* diverge as *T* 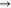 0), the quasicrystal manifests strong quantum critical effects (*ρ* ∼ *T* and both 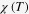 and 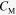/*T* diverge as *T* 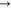 0). This behavior is unchanged for the quasicrystal under hydrostatic pressure but modified under an applied magnetic field. Similar studies by Watanuki *et al* [101], however, found evidence of quantum critical behavior in both the quasicrystalline and approximant phases. Clearly, further studies of the quantum critical behavior in these alloys are called for.

## 4. Binary magnetic icosahedral quasicrystals and approximants in *R*-Cd

**Figure 11.**
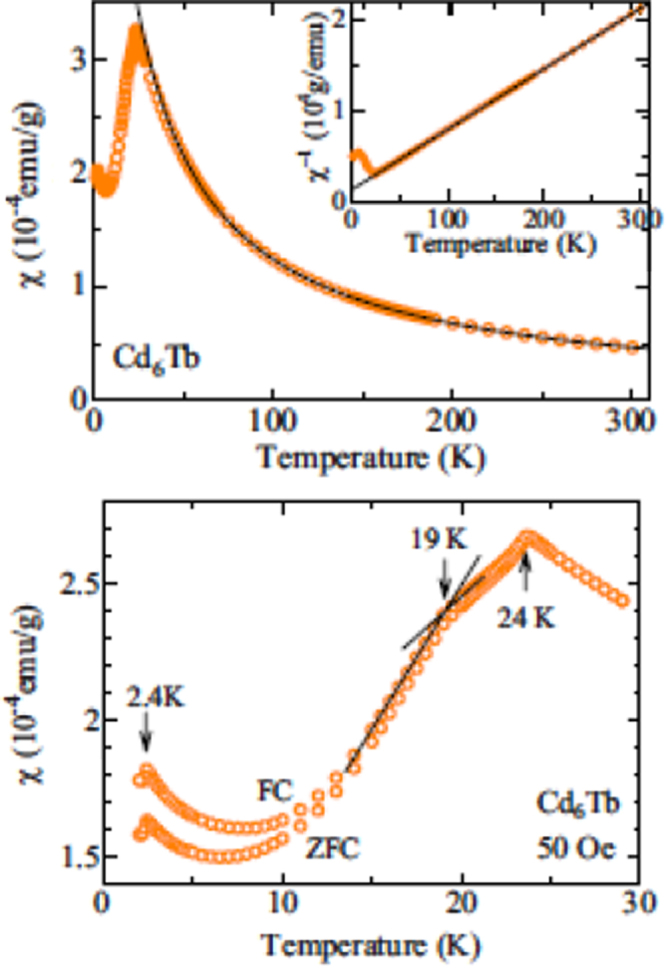
(Top) Magnetic susceptibility of TbCd_6_ from 1.8 to 300 K measured under a magnetic field of 1000 Oe. The solid line is a fit to the Curie–Weiss law between 50 and 300 K. The inset shows the inverse susceptibility of TbCd_6_. (Bottom) Magnetic susceptibility of TbCd_6_ at low-temperature from 1.8 to 30 K measured under a magnetic field of 50 Oe. The curve displays two anomalies at 24 and 2.4 K and a kink at 19 K as denoted by the arrows. FC and ZFC magnetic susceptibilities are shown. After [47].

Another important development in the investigation of magnetism in quasicrystals was the discovery of AFM order in the *R*Cd_6_ cubic approximants, first indicated by bulk magnetization measurements [47, 49, 50] and established to be long-range AFM order by x-ray resonant magnetic scattering (XRMS) measurements at the Tb 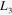 x-ray absorption edge of a single crystal 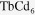 [48]. Figure 11 shows the magnetic susceptibility of 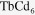 which exhibits anomalies at 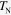 = 24 K, 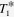 = 19 K and 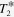 = 2.4 K [47]. Interestingly, the FC and ZFC curves separate below 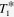 and the 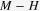 curves at the lowest temperature (2 K) also exhibits hysteresis between the magnetization and demagnetization curves. These features point to the possibility that not all Tb moments participate in the AFM order and, instead, freeze at lower temperature. Mori *et al* have presented an extensive study of the magnetic properties of the *R*Cd_6_ approximants for *R* = Y, Pr, Nd, Sm and Gd through Lu [49]. All of the heavy magnetic rare earths manifest at least one, and often several anomalies in magnetic susceptibility, resistivity and specific heat that may be associated with AFM ordering and/or additional magnetic transitions. A summary of the magnetic properties of the *R*Cd_6_ approximants is provided in table 2,2.

**Table 2.** Magnetic properties of the binary *R*Cd_6_ cubic approximants. 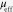 is the measured effective moment per *R*-ion, 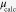 is the calculated free-ion value, 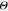 is the measured Weiss temperature, and 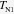 through 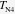 refer to temperatures where anomalies in bulk transport and thermodynamic measurements suggest magnetic ordering transitions.

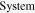	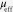	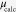	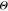	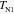	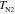	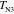	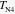	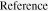
	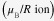	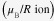	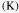	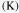	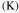	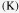	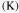
PrCd_6_	3.65	3.58	−11.33	0.13	—	—	—	[49]
NdCd_6_	3.55	3.62	−5.75	4.8	2.5	—	—	[49]
SmCd_6_	—	0.84	—	12.2	9.0	5.7	—	[49]
	0.59	0.84	−16	12.5	10.2	6.5	—	[47]
GdCd_6_	7.94	7.94	−32	18.9	13.2	7.3	2.5	[49]
TbCd_6_	9.3	9.72	−18	22.4	17.6	—	—	[49]
	9.8	9.72	−17	24	19	2.4	—	[47]
DyCd_6_	10.9	10.63	−5.1	17.8	—	—	—	[49]
HoCd_6_	10.5	10.60	−1.0	8.4	6.8	3.4	—	[49]
ErCd_6_	9.1	9.59	−0.9	2.8	—	—	—	[49]
TmCd_6_	7.4	7.57	−3.1	2.2	—	—	—	[49]

**Figure 12.**
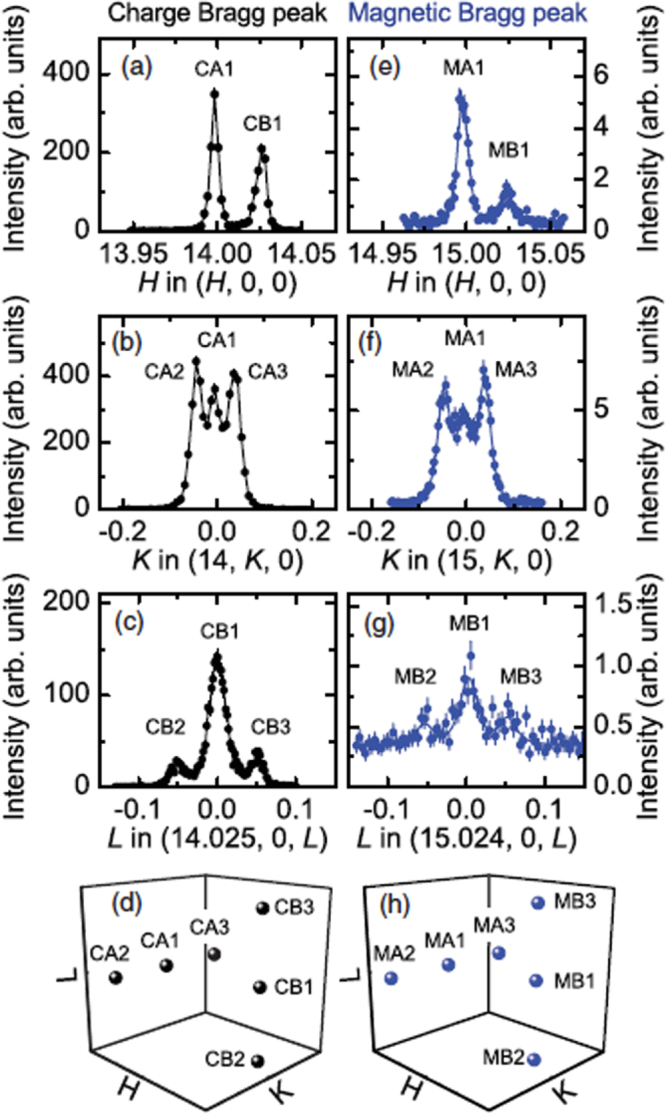
Distribution of charge and magnetic peaks measured by an (a) *H* scan, (b) *K* scan, and (c) *L* scan through the (14 0 0) charge peak position and an (e) *H* scan, (f) *K* scan, and (g) *L* scan through the (15 0 0) magnetic peak position. Panels (d) and (h) plot the charge and magnetic peak positions schematically. After [48].

The XRMS measurements on 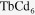 [48] and 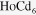 [51] clearly demonstrated that long-range AFM order is established in these approximants below 24 K and 8.5 K, respectively. Shown in figure 12 are scans through the charge peak and magnetic peak positions showing that sharp Bragg peaks are observed for the magnetic scattering from 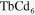 with a magnetic correlation length greater than 500 Å. The structure observed for the charge and magnetic peaks arises from the different domains associated with the cubic-to-monoclinic structural transition at much higher temperature [58]. This domain structure presents both a blessing and a curse for the ultimate determination of the magnetic structure of the *R*Cd_6_ approximants. The low symmetry of the monoclinic phase should permit a full determination of not just the ordering-type, but the moment directions as well. On the other hand, a complete determination of the monoclinic domain distribution for sample must first be accomplished.

**Figure 13.**
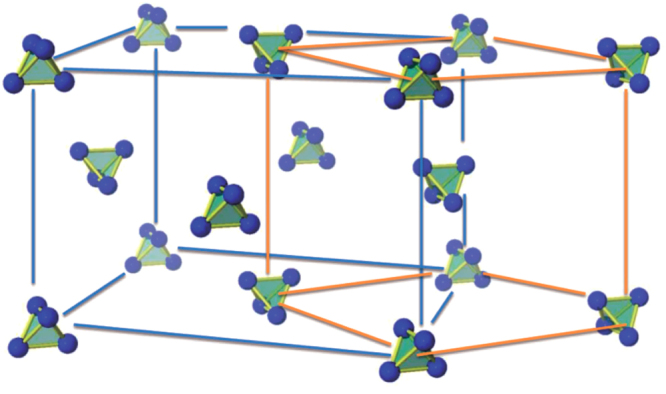
Low-temperature superstructure of *R*Cd_6_ showing the high-temperature cubic (orange lines) and low-temperature monoclinic (blue lines) unit cells, and the orientational ordering of the Cd tetrahedra within the Tsai clusters. After [58].

In terms of the high-temperature cubic unit cell and body-centered arrangement of *R* icosahedra (see figure 1), the AFM order breaks the body-centering translational symmetry of the chemical unit cell such that the Tb ions associated with the icosahedral cluster at the corner of the unit cell are antiferromagnetically correlated with the Tb ions associated with the icosahedral cluster at the center of the unit cell. Very recent neutron diffraction measurements on ^112^Cd isotope enriched single grains of TbCd_6_ , however, have shown that the AFM order is best described in terms of the monoclinic unit cell by a magnetic wavevector of (1 0 0) [105]. This is quite interesting because the magnetic structure, then, follows the pattern of ordering of the inner tetrahedra of Cd atoms within the Tsai clusters, shown in figure 13, that drives the structural distortion at higher temperature [58]. No clear evidence of additional magnetic transitions in TbCd_6_ at lower temperatures, corresponding to the anomalies in the magnetic susceptibility at 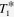 = 19 K and 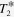 = 2.4 K, were observed in the XRMS and neutron diffraction measurements leaving the origin of these features as an open question.

Very recently, the corresponding binary *R*-Cd icosahedral quasicrystals were discovered [46] using the strategy developed in the identification of a binary quasicrystal in the Sc-Zn system [106]: binary quasicrystalline phases may well exist nearby known approximants, perhaps as peritectically forming compounds with very limited liquidus surfaces, offering very limited ranges of composition/temperature for primary solidification. One interesting observation regarding these binary phases is that their compositions range from GdCd_7.9_ to TmCd_7.3_ differing significantly from the prototypical YbCd_5.7_ icosahedral quasicrystal and RCd_6_ cubic approximants, but closer to the stoichiometry of the Sc_12_ Zn_88_ and *R*-Mg-Cd quasicrystals. This suggests that these binary quasicrystals may comprise a new sub-class of the Tsai-type icosahedral phases.

**Figure 14.**
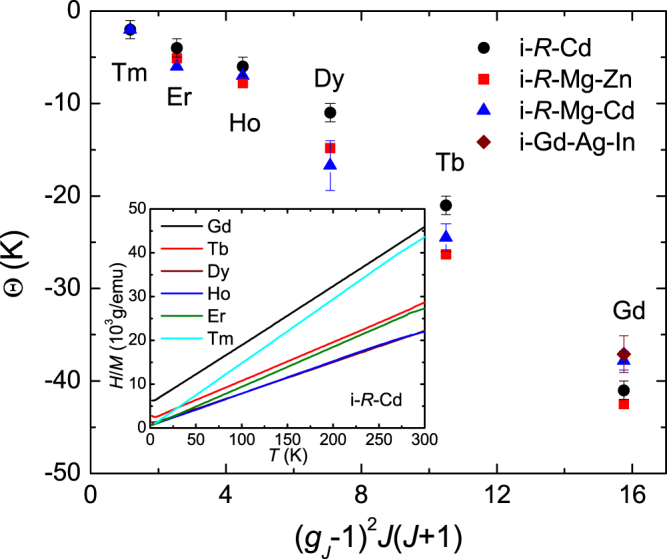
Weiss temperature values versus de Gennes factor. Solid points represent the Weiss temperature, 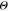, values for the *R*-Cd quasicrystals (black circles) obtained in the present measurements from a linear fit of the high-temperature inverse magnetic susceptibility measured at either *H* = 5000 or 10,000 Oe (shown in the inset). After [46].

**Figure 15.**
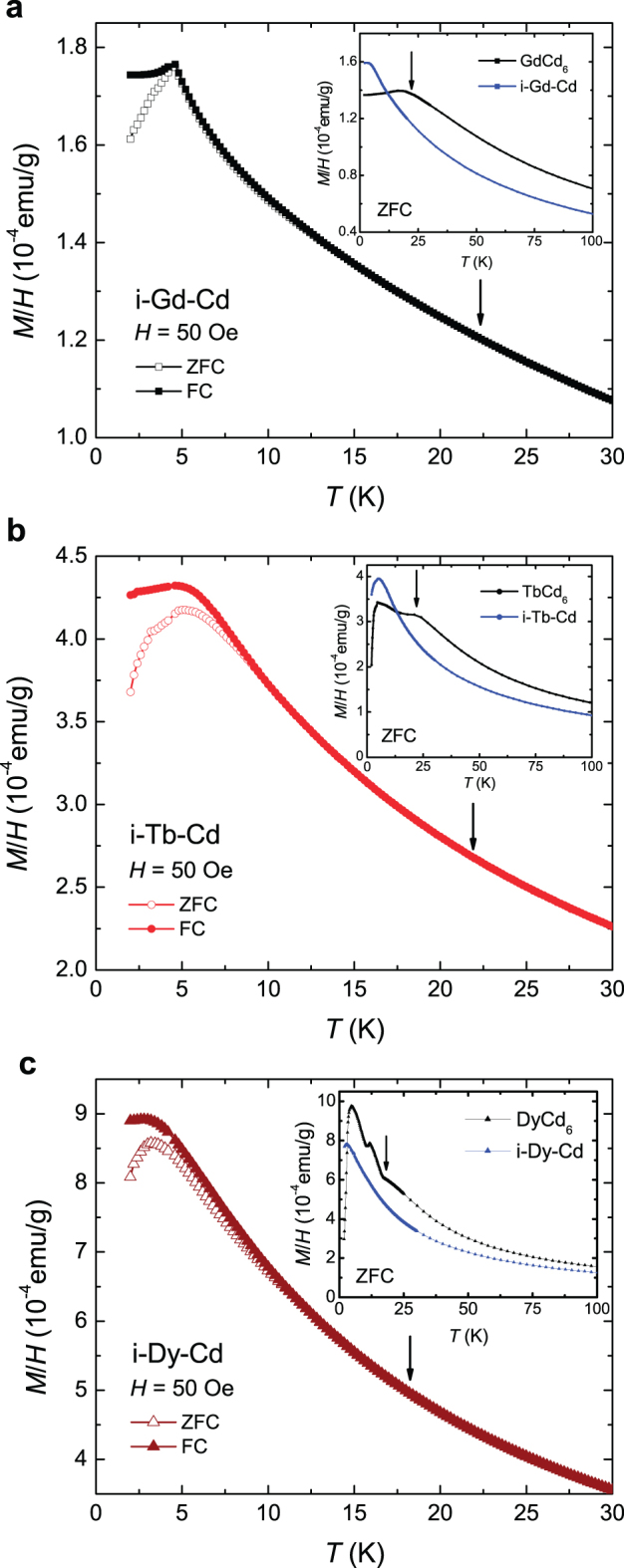
Temperature-dependent FC and ZFC magnetization data measured under a field of 50 Oe from (a) Gd-Cd, (b) Tb-Cd and (c) Dy-Cd. The insets compare the higher-field magnetization data (ZFC) for icosahedral phase samples with their respective *R*Cd_6_ approximants. The arrows in the insets indicate the location of clear magnetic ordering features for the approximants, which are absent for the related *R*-Cd quasicrystalline compounds (main figure). After [46].

The magnetic properties of the *R*-Cd binary quasicrystals are, in fact, quite similar to what has been discussed for the ternary *R*-containing system and different from what was found for the *R*Cd_6_ cubic approximants (see table 3). At high temperatures, the inverse magnetic susceptibilities of the *R*-Cd quasicrystals are isotropic and closely follow the Curie–Weiss law with effective moments that are consistent with the free 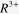 values. The Weiss temperatures derived from fits using the Curie–Weiss law are negative, indicating primarily AFM interactions between the *R* ions. In fact, the similarity in the values for 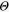, shown in figure 14 for the *R*-Cd, *R*-Mg-Zn, *R*-Mg-Cd and Gd-Ag-In quasicrystals, suggest that that the strength of the AFM exchange is quite similar for all series. At low-temperature, a cusp-like feature associated with a spin-freezing temperature (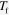) is observed in the zero-field-cooled (ZFC) magnetic susceptibility and, similar to the *R*-Mg-Cd ternary systems, for some members of this series (e.g. Tb and Dy) the maximum in *χ* appears below the bifurcation of the FC and ZFC curves. Whereas the *R*Cd_6_ cubic approximants manifest long-range magnetic order below 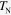, the *R*-Cd quasicrystals a SG-like transition (figure 15) at values of 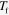 comparable to other *R*-containing quasicrystals.

**Table 3.** Magnetic properties of the binary *R*-Cd icosahedral quasicrystals. 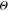 is the measured Weiss temperature and 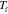 is the spin freezing temperature determined from the cusp in the zero-field-cooled dc magnetization data. After [46, 107].

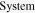	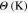	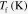
GdCd_7.88_	−41	4.6
TbCd_7.69_	−21	5.3
DyCd_7.50_	−11	3.0
HoCd_7.60_	−6	1.76
ErCd_7.34_	−4	1.11
TmCd_7.28_	−2	0.63

## 5. Summary

Taken all together, the experimental studies reviewed here paint a complex picture of magnetism in the *R*-containing icosahedral quasicrystals. The absence of long-range magnetic order and the presence of spin-glass-like freezing of the moments at low temperature seems almost ubiquitous to this class of compounds. The neutron data, in particular, point to the predominance of short-range magnetic interactions and magnetic clustering consistent with cluster-based structural models for the icosahedral phase and site-ordered geometrically frustrated magnetism. Magnetic frustration can arise from at least two sources here: (i) the geometry of the clusters themselves which feature pentagonal arrangements (in the case of the dodecahedral clusters for *R*-Mg-Zn) or triangular arrangements (in the case of the Tsai-type clusters) of moments; and (ii) the quasiperiodic arrangement of the clusters in the icosahedral quasicrystal. However, particularly for the ternary systems, even if the *R* sites are well defined (at least for the majority of the *R* ions) one should not overlook the effects of chemical and/or topological disorder that can arise from some degree of randomness in the nominal Cd-site occupancies for Mg and Zn, or Mg and Cd. In short, the *R*-containing magnetic quasicrystals might best be classified as intermediate between canonical SG and superparamagnets, showing elements of both.

Clearly, several issues remain unresolved. First and foremost, it is not clear why the static short-range magnetic order found in both elastic and inelastic scattering measurements, do not propagate beyond individual clusters. Is this a property or result of quasiperiodicity? Secondly, the role of the CEF, which generally produces local moment anisotropy, remains somewhat mysterious. Investigations by Fisher *et al* [73] of a series of (Y_1−*x*_ Tb_*x*_ )-Mg-Zn and (Y_1−*x*_ Gd_*x*_ )-Mg-Zn phases find that local moment anisotropy caused by CEF effects play a significant role in increasing 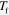, but no distinct CEF excitations have been observed in inelastic neutron scattering measurements on quasicrystals. Finally, the inelastic neutron scattering measurements show a wide range of microscopic behavior. In particular, the low-energy excitation spectra are very different for Tb-Mg-Zn and Ho-Mg-Zn implying very different spin dynamics in these quasicrystals, the origin of which is unknown.

For the new *R*-Cd binary phases several new investigations are suggested by the data. For example, the difference in composition between the *R*-Cd quasicrystals and the YbCd_5.7_ quasicrystal, as well as the *R*Cd_6_ quasicrystal approximants, raises the question of whether there are fundamental differences between the structures of these systems. It has been argued [3, 46] that in the quasicrystalline phase the *R* icosahedra remain intact, but the glue filling the gaps between the Tsai clusters is deficient in *R* ions. This is substantiated by the fact that the measured *R*Cd_6_ lattice constants are very close to the value calculated for the 1/1 cubic approximant from the measured 6D lattice constant [46]. Nevertheless, a full 6D structural refinement of the *R*-Cd quasicrystal, as was done for YbCd_5.7_ , would be highly desirable to confirm this proposal.

Ultimately, for these new binary phases, we are left with the question of why long-range AFM order is established in the approximant phases and not in the quasicrystals. A comprehensive study of the structure and magnetic properties of these systems may well provide new insights into the impact of quasiperiodicity on magnetism. Finally, the search for new magnetic quasicrystals is ongoing, stimulated, in part, by novel behavior in quasicrystals like Yb-Au-Al and the prospect of discovering the first quasiantiferromagnet.
